# Study of Nephrotoxic Potential of Acetaminophen in Birds

**DOI:** 10.4103/0971-6580.72677

**Published:** 2010

**Authors:** K. Jayakumar, K. Mohan, H. D. Narayana Swamy, N. B. Shridhar, M. D. Bayer

**Affiliations:** Department of Veterinary Pharmacology and Toxicology, Veterinary College, KVAFSU, Hebbal, Bangalore, India; 1 Department of Veterinary Pathology, Veterinary College, KVAFSU, Hebbal, Bangalore, India; 2 Department of Veterinary Faculty Clinics, Ningarhar University, Daronta Jalalabad, Ningarhar, Afghanistan

**Keywords:** Acetaminophen, birds, diclofenac, nephrotoxicity

## Abstract

The present study was designed to evaluate the effect of acetaminophen on kidneys of birds by comparison with diclofenac that is used as positive control. The birds of Group I served as negative control and received normal saline, whereas Group II birds received diclofenac injection (2.5 mg/kg IM) and Group III birds received acetaminophen injection (10 mg/kg IM) for a period of seven days daily. The birds treated with diclofenac showed severe clinical signs of toxicity accompanied with high mortality and significant increase (*P*<0.001) in serum creatinine and uric acid concentration. The creatinine and uric acid concentrations were consistent with gross and histopathological findings. The negative control and acetaminophen-treated groups showed no adverse clinical signs, serum creatinine and uric acid concentrations were normal, and no gross or histopathological changes in kidneys were observed. Thus, it was concluded that acetaminophen can be used for treatment in birds without any adverse effect on kidneys.

## INTRODUCTION

Nonsteroidal antiinflammatory drugs (NSAIDs) are extensively used in human medicine and there are numerous reports of toxicity in humans. Nephrotoxicity is one of the important adverse effect reported very frequently.[1] NSAIDs apart from their use in domestic animals are also used in birds for treatment. Diclofenac has been well documented as a nephrotoxic drug in the birds[2,3] and vultures.[4] Diclofenac was attributed as major cause for decline in vulture population across Indian sub-continent. Recently, acetaminophen (*N*-acetyl-p-aminophenol), also known as paracetamol, has been introduced to veterinary use for the treatment of domestic animals. Veterinarians may use acetaminophen for treatment in birds. In view of the premise that NSAIDs are potentially nephrotoxic in avian species, the present study was designed to investigate the potential of acetaminophen to cause nephrotoxicity in comparison with diclofenac in broiler chickens.

## MATERIALS AND METHODS

A total of 18 apparently healthy broiler birds aged five weeks with body weight ranging from 1.5 to 1.8 kg were procured from a commercial broiler farm. The birds were caged individually in experimental animal house with standard laboratory conditions and were allowed to acclimatize to the laboratory housing condition for a period of five days. Medication-free feed procured from University poultry farm was fed *ad libitum* along with free access to potable water.

The broiler chickens were randomly divided into three groups with six birds in each. The birds of group I served as normal control and received normal saline IM, whereas group II birds received diclofenac injection (2.5 mg/kg IM) and group III birds received acetaminophen injection (10 mg/kg IM) for a period of seven days. All the birds were observed for clinical signs of illness, if any, mortality was recorded, necropsy was conducted on dead birds, and tissue samples were collected for histopathology. Approximately 1 ml of blood was collected through wing vein using sterile disposable syringe and needle at 0 hour (before treatment) and subsequently at an interval of 24 hours for seven days. The serum was separated and used for estimation of creatinine and uric acid concentration, using commercial diagnostic kits (M/s. Swemed diagnostics, Bangalore) and ARTOS semiautomatic biochemical analyzer.

At the end of the experiment, all the surviving birds were sacrificed. Systematic postmortem examinations were conducted and gross lesions, if any, were recorded. The representative tissue samples from kidney were collected in 10% neutral buffered formalin and in absolute alcohol. The tissues fixed in neutral buffered formalin were processed by routine paraffin embedding technique and sections of 5 *µ* were cut. These sections were stained using routine hematoxylin and eosin. The tissues which were fixed in absolute alcohol were directly cleared and infiltrated with paraffin, and sections were stained by De-Galantha’s method to demonstrate urate crystals.[5]

The data were recorded and analyzed for their significance using student’s *t* test employing GraphPad Prism[6] computer software.

## RESULTS AND DISCUSSIONS

In the present study, the diclofenac-administered birds showed clinical manifestations such as anorexia, dullness, ruffled feathers, lethargy, depression, recumbence, sunken eyes, and watery droppings on day 2 and was continued to be so till the end of experiment. Similar clinical signs were also observed by Swetha *et al*.[2] in birds administered with diclofenac and in natural cases of gouty birds.[7] However, no such clinical manifestations were observed in other groups. The clinical signs observed in the diclofenac-treated group can be attributed to potential toxicity of diclofenac on kidneys.

The diclofenac-treated birds showed mortality on day 3, 4, and 6. Of the six birds, four died during the experimental period. Similarly, high mortality has been reported in the birds which were administered with diclofenac.[2,3] In contrary, mortality was not observed in broiler birds administered with high dose of meloxicam[2] and in the birds exposed to nimesulide.[2,3] Furthermore, in the present study, mortality was not observed in the birds administered with acetaminophen. The high mortality due to diclofenac can be attributed to acute renal failure leading to visceral gout, which was evident both grossly and microscopically in the present investigation.

Serum biochemical analysis showed a gradual but significant increase (*P*<0.001) in creatinine and uric acid from day 2 onwards in diclofenac-treated birds [Table 1]. Similarly, the significant increase in serum uric acid and creatinine concentration in birds treated with diclofenac has been well documented.[2,3] Increase in serum uric acid concentration has also been reported in natural cases of visceral gout.[8] An increased serum uric acid and creatinine concentration can be attributed to impaired uric acid excretion due to tubular degeneration caused by diclofenac, leading to renal failure in birds. This could result in accumulation of uric acid in blood (hyperuricemia) and tissues leading to visceral gout, which might be responsible for high mortality in birds.

**Table 1:** Serum creatinine and uric acid concentration in control, diclofenac- and acetaminophen-treated group

Day	Group I (control)		Group II (diclofenac)		Group III (acetaminophen)	
	Creatinine (mg/dl)	Uric acid (mg/dl)	Creatinine (mg/dl)	Uric acid (mg/dl)	Creatinine (mg/dl)	Uric acid (mg/dl)
1	0.38 ± 0.02	2.53 ± 0.09	0.38 ± 0.03	2.47 ± 0.13	0.42 ± 0.04	2.37 ± 0.07
2	0.43 ± 0.03	2.56 ± 0.06	0.62 ± 0.03*	4.41 ± 0.22*	0.38 ± 0.02	2.54 ± 0.06
3	0.37 ± 0.03	2.63 ± 0.06	0.73 ± 0.03* n = 5	6.36 ± 0.15* n = 5	0.37 ± 0.01	2.56 ± 0.05
4	0.42 ± 0.02	2.75 ± 0.11	0.83 ± 0.02* n = 3	6.87 ± 0.33* n = 3	0.38 ± 0.02	2.83 ± 0.03
5	0.42 ± 0.03	2.83 ± 0.10	0.84 ± 0.02* n = 3	8.29 ± 0.33* n = 3	0.38 ± 0.01	2.90 ± 0.05
6	0.42 ± 0.03	3.14 ± 0.06	0.85 ± 0.03* n = 2	8.96 ± 0.44* n = 2	0.40 ± 0.02	2.99 ± 0.07
7	0.44 ± 0.02	3.11 ± 0.02	0.93 ± 0.01* n = 2	10.20 ± 0.36* n = 2	0.50 ± 0.01	3.26 ± 0.04

n = 10, unless otherwise mentioned; Values are in Mean ± SE,

* *P* < 0.001

The birds in control and acetaminophen group showed no significant change in the serum creatinine and uric acid concentration.

**Figure 1:**
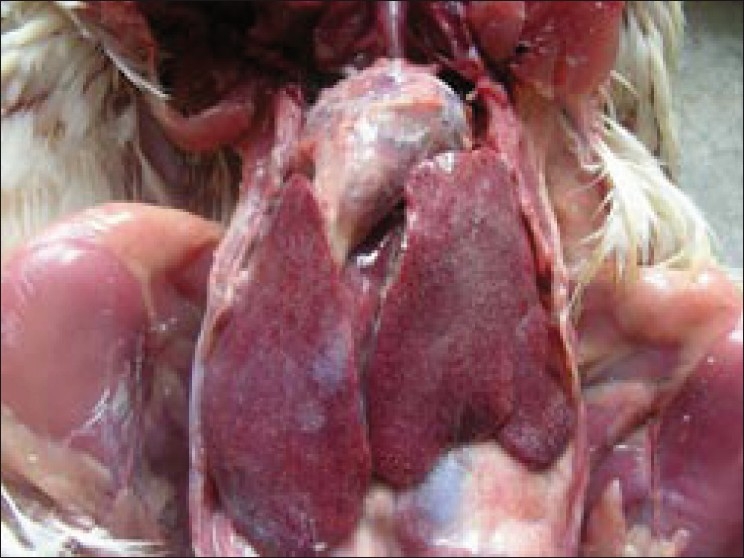
Diclofenac-treated (Group II) bird showing congested musculature along with deposition of grayish white urates on the visceral organs

**Figure 2:**
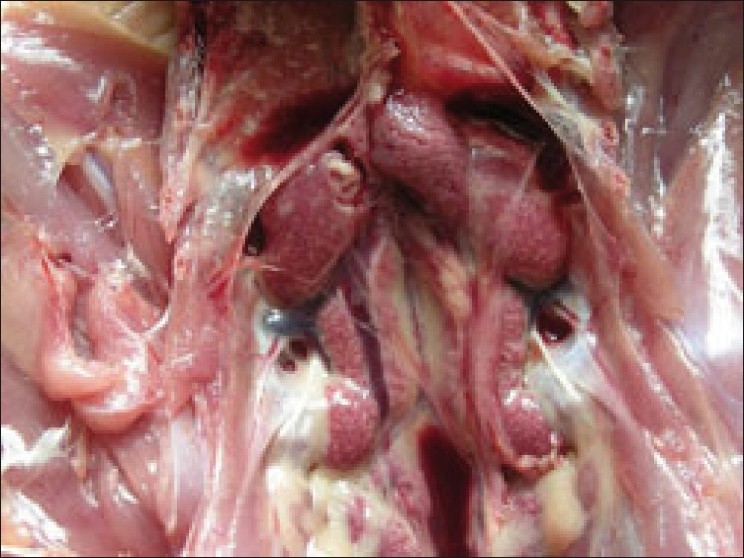
Kidney (Group II) showing enlargement with prominent lobulation and deposition of urate crystals

Postmortem examination of the birds treated with diclofenac revealed congested musculature along with deposition of grayish white urates on the visceral organs [Figure 1]. The kidneys were whitish grey in appearance and were considerably enlarged with bulging out of the renal fossa [Figure 2]. Focal or multifocal chalky white urate crystal deposits were noticed on the kidneys. However, no such lesions in the kidney were observed in control group and in birds treated with acetaminophen.

Similar gross lesions were consistently observed in the kidney of birds administered with diclofenac[2] and in birds died due to visceral gout.[7] The swollen kidneys with prominent lobules may be due to marked accumulation of urates in the tubules along with degenerative changes. Uric acid is the end product of purine metabolism produced normally in the healthy birds, and hyperuricemia along with renal malfunction may lead to gout, where in uric acid get accumulated in the kidney tubules resulting in renal failure.[9]

**Figure 3:**
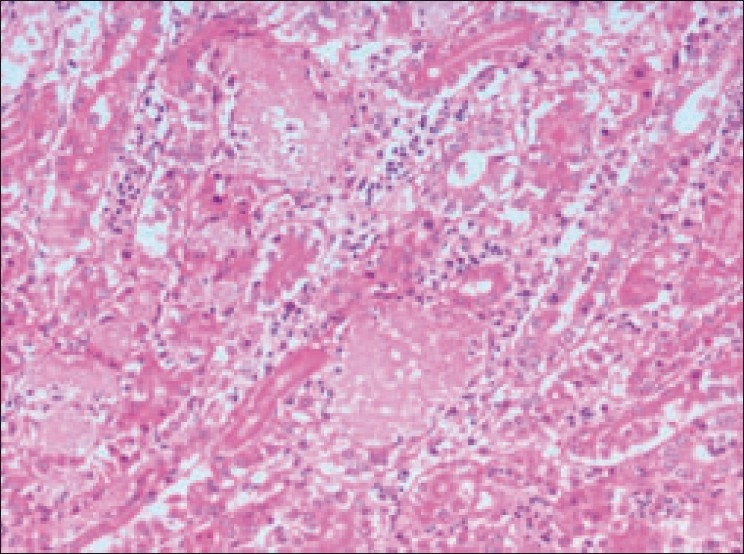
Section of kidney (Group II) showing degeneration and necrosis of tubular epithelium with infiltration of inflammatory cells. Note the areas of urate crystal deposition appearing as homogenous pinkish areas or clefts (H and E, ×500)

On microscopic examination, in the diclofenac-treated group, kidney section showed vascular and degenerative changes along with urate deposition. Focal or multifocal areas of tubular epithelial degeneration and necrosis accompanied with infiltration of inflammatory cells [Figure 3]. In concurrence with the present findings, numerous large aggregates of amorphous urate material and cell debris with infiltration of inflammatory cells, accompanied with loss of normal renal architecture, were prominent in kidney sections of birds died after experimental diclofenac toxicity.[2,3] Furthermore, such lesions were also reported in birds which died due to visceral gout.[7]

**Figure 4:**
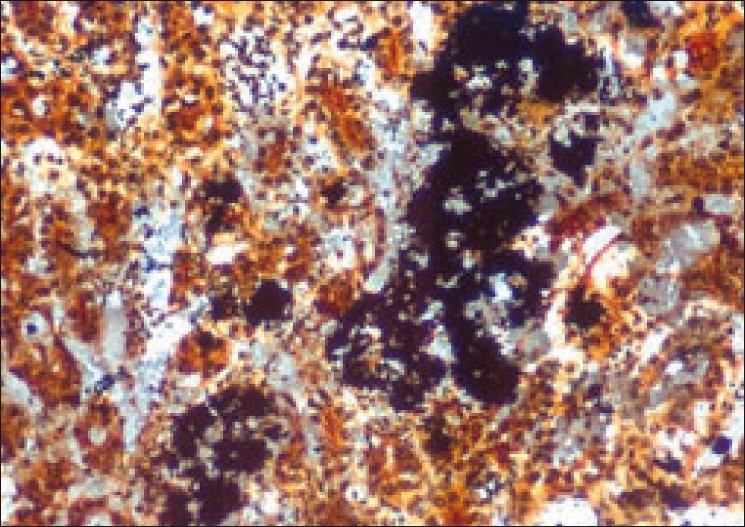
Section of kidney (Group II) showing deposition of black-stained urate crystals in renal tubules (Degalanthas stain, ×500)

In diclofenac-treated group, deposition of uric acid crystals in clumps [Figure 4] with occasional focal irregular black spots were very commonly noticed in the De-Galantha’s stained sections.

**Figure 5:**
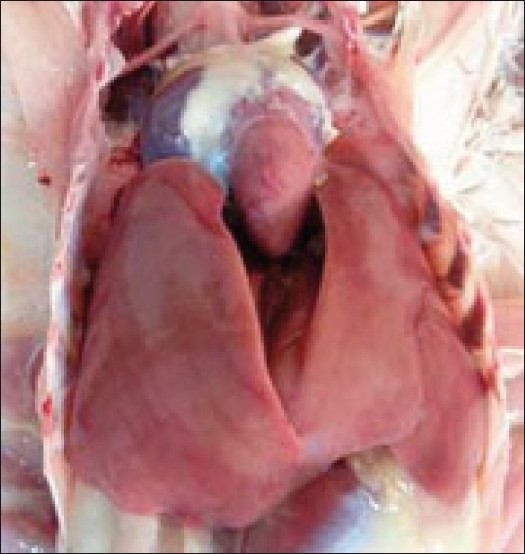
Acetaminophen-treated (Group III) bird showing no apparent gross lesions

**Figure 6:**
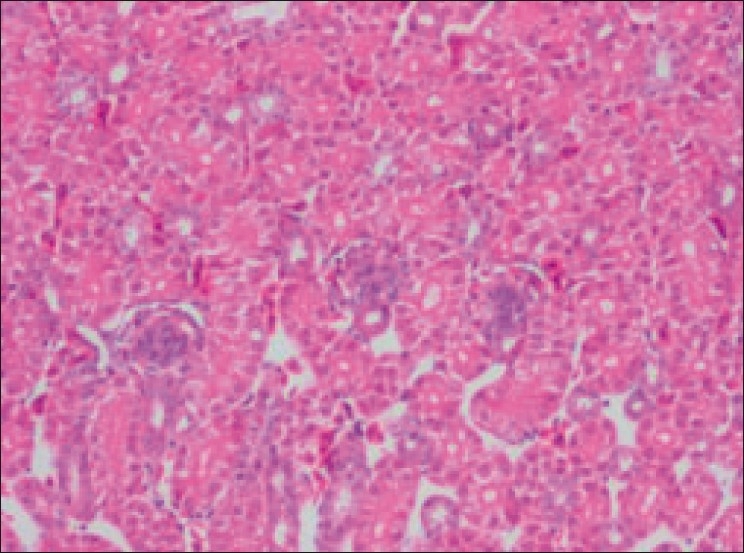
Section of kidney–group III–showing mild hemorrhage and apparently normal architecture of the kidney (H and E, ×500)

No apparent gross lesions or histopathological lesions were observed in the kidney sections of birds in control and in the birds treated with acetaminophen [Figures 5 and 6].

## CONCLUSION

In the present study, acetaminophen-treated group showed no adverse clinical signs. Serum creatinine and uric acid concentration was normal and no gross and histopathological changes in kidneys were observed. It was concluded that acetaminophen does not cause any nephrotoxicity in birds like diclofenac and it can be used safely in treatment of birds.
